# The Giant Anteater in the Room: Brazil's Neglected Tropical Diseases Problem

**DOI:** 10.1371/journal.pntd.0000177

**Published:** 2008-01-30

**Authors:** Peter J. Hotez

**Affiliations:** Sabin Vaccine Institute and Department of Microbiology, Immunology, and Tropical Medicine, George Washington University Medical Center, Washington, D. C., United States of America

The phrase “the 800-pound gorilla in the room” refers to an obvious problem that everyone knows exists but pretends or chooses to ignore. In my December 2007 *PLoS Neglected Tropical Diseases* editorial I wrote about an unseemly underbelly of poverty that exists in my country, the United States of America, and the unacceptable disease burden among our poor that results from such neglected diseases as toxocariasis, cysticercosis, and toxoplasmosis [1]. According to the Pan American Health Organization (PAHO) (the regional office of the World Health Organization in the Americas) the Latin American and Caribbean region suffers from much larger pockets of poverty, and with it endemic neglected tropical diseases (NTDs) [2]. While researching a review of the NTDs in Latin America, I was particularly struck by the disproportionate concentration of these conditions among the poor living in Brazil. Although there are no gorillas in the rooms of Brazil I have concluded that the NTDs represent an ominous giant anteater (*Myrmecophaga tridactyla*, the largest species of anteater found in Brazil and elsewhere in the American tropics) that requires notice, attention, and urgent action.

According to the World Bank, 22% of the population of Latin America and the Caribbean lives on less than US$2 per day [3]. An identical percentage of Brazil's population is also impoverished [4]. However, based on the Gini coefficient index, a metric that measures inequality of income or wealth distribution, Brazil at 0.59 has one of the greatest disparities between wealthy and poor people anywhere in the world [5]. One of the ways that this inequality manifests is in a shockingly high burden of NTDs. The roughly 40 million *Brasileiros* who live on less than US$2 per day [4] account for one-third of all of the poor people living in the Latin American and Caribbean region [3], but they suffer disproportionately from NTDs. Shown in Table 1 is a summary of Brazil's NTD burden. Based on information available from published peer-reviewed papers in the biomedical literature, as well as publicly available Web sites from PAHO and WHO [6]–[13], most of the NTD disease burden in Latin America and the Caribbean now occurs in Brazil, including virtually all of the cases of blinding trachoma and leprosy, and the majority of ascariasis, dengue, hookworm infection, schistosomiasis, and visceral leishmaniasis. Practically speaking these data mean that most of Brazil's poorest 40 million people are infected with one or more NTD, especially either hookworms (*Necator americanus*) or roundworms (*Ascaris lumbricoides*) or both. For instance, in collaboration with the Centro des Pesquisas Rene Rachou of FIOCRUZ (Fundacao Oswaldo Cruz) we recently showed that 68% of some rural communities in Minas Gerais State are infected with hookworms [14], which cause anemia and malnutrition [15]. In these same poor people the presence of hookworms in their intestines promotes susceptibility to coinfections with other worms, especially *Ascaris* roundworms and schistosomes (14).

**Table 1:** Burden of Neglected Tropical Diseases in Brazil

Disease	Percentage of Latin America's Disease Burden that Occurs in Brazil	Estimated No. Cases in Brazil	Reference
Blinding trachoma	97%	1.06 million	[6]
Leprosy	93%	44,436 new cases (2006)	[7]
Schistosomiasis	83%	1.5 million	[8]
Visceral leishmaniasis	67%	3,386 (2004)	[9]
Hookworm infection	65%	32.3 million	[10]
Dengue	63%	346,471 reported cases (2006)	[11]
Ascariasis	50%	41.7 million	[10]
Cutaneous leishmaniasis	46%	28,375 (2004)	[9]
Trichuriasis	19%	18.9 million	[10]
Lymphatic filariasis	8%	60,000	[12]
Onchocerciasis	2%	9,000 at risk	[2]
Leptospirosis	Not determined	Not determined	[13]

Throughout Minas Gerais and neighboring Bahia states, hookworm is known as *amarelao*, referring to the yellow–sallow complexion that results from chronic infection. Hookworm and other important NTDs are also major public health problems among Brazil's indigenous people [16]–[19] and citizens of African descent [20]. Recently, an economist at the University of Chicago found that chronic hookworm infection during childhood reduces future wage-earning by 43% [21] meaning that hookworm represents an important reason why poor people become trapped in poverty. This is the very same hookworm that sapped the energy from Jeca Tatu, the laborer popularized by the Brazilian writer Monteiro Lobato in the early twentieth century. An important feature of almost all of the NTDs is that they not only occur in the poverty setting but also promote poverty [22].

In 2003, President Luiz Inacio Lula a Silva launched *Zero Hunger*, an ambitious $500 million antipoverty drive focused on malnutrition [23]. However, as the World Food Programme has already discovered [24], in the absence of concurrent NTD control, measures that feed the children often simply result in feeding the worms first. Concurrent NTD control would make great sense, as it is now ranked among the most cost-effective health measures [22], and at a 15%–30% rate of return, a highly cost-effective antipoverty measure as well [25],[26]. NTD control would represent one of the most efficient and effective mechanisms for lifting the 40 million most poor Brazilians out of poverty.

Everything is in place for Brazil to launch a nationwide effort geared at solving one of its greatest health disparities. The country has a charismatic president who is committed to the poor; it has in FIOCRUZ and other health ministry agencies and its universities some of the best disease control experts anywhere in the world (many of whom serve on the Editorial Board of *PLoS Neglected Tropical Diseases*); and, through these same organizations and a sophisticated biotechnology manufacturing infrastructure at Instituto Butantan (Sao Paulo) and Bio-manguinhos (Rio de Janeiro), the ability to innovate and produce a new and improved generation of “antipoverty” vaccines for NTDs. These vaccines target dengue, hookworm, leishmaniasis, leptospirosis, schistosomiasis, and yellow fever [27],[28]. Together with new foundations committed to solving health and other disparities in the Americas and some of the highest net-worth individuals anywhere, Brazil is in an excellent position to establish vital public–private partnerships to control, or in some cases, eliminate its most important NTDs. It has already taken leadership in the elimination of its Chagas disease problem, formerly one of the most devastating NTDs in the Southern Cone of South America [29], and has made great strides in the control and elimination of lymphatic filariasis and onchocerciasis [2],[12]. Now, just as Jeca Tatu who, once cured of his hookworm, championed social change, so too a new Brazilian public–private partnership could one day eliminate its NTDs as a substantive means for reducing poverty and achieving important Millennium Development Goals (an international set of goals and targets for sustainable poverty reduction in developing countries by the year 2015). As we enter the season of *Carnaval* this is a special time to remember Brazil's poorest 40 million people. In the meantime, *PLoS Neglected Tropical Diseases* remains committed to receiving and reviewing papers on the NTDs of Brazil and helping to facilitate communication among the Brazilian scientific community.

## Supporting Information

Text S1 Portuguese Translation of the Article by Helton Santiago
(0.07 MB DOC)
