# Supplementary material for: The Giant Anteater in the Room: Brazil's Neglected Tropical Diseases Problem
Source: PLoS Negl Trop Dis. 2008 Jan 30;2(1):e177. doi: 10.1371/journal.pntd.0000177 (PMC2265550; doi:10.1371/journal.pntd.0000177)
Supplement: Text S1 — Portuguese Translation of the Article by Helton Santiago (0.07 MB DOC) [file pntd.0000177.s001.doc]

*PLoS Neglected Tropical Diseases Editorial*

**O Tamanduá Gigante na Sala**

*O Problema das Doenças Tropicais Negligenciadas do Brasil*

Peter Hotez*

Sabin Vaccine Institute and Dept. of Microbiology, Immunology, & Tropical Medicine,

The George Washington University, Washington, D.C., United States of America

**Financiamento**: O autor não recebeu nenhum financiamento específico para esse estudo

**Conflito de interesse**: PH é Diretor Executivo da Rede Global para Doenças Tropicais Negligenciadas e Presidente do Sabin Vaccine Institute. Ele é inventor de duas patentes internacionais em vacina para ancilostomídeos.

**Informações sobre o autor**: Peter Hotez, MD, PhD é Editor-Chefe da *PLoS Neglected Tropical Diseases*, além de Professor do Walter G. Ross e Chefe do Departamento de Microbiologia, Imunologia e Medicina Tropical da George Washington University e Presidente do Sabin Vaccine Institute

*Email: [mtmpjh@gwumc.edu](mailto:mtmpjh@gwumc.edu) or [photez@gwu.edu](mailto:photez@gwu.edu)

A frase “um gorilla de 800 libras na sala” refere-se a um problema óbvio que todos sabem da existência, mas fingem ou escolhem ignorá-lo. Em meu editorial de Dezembro de 2007 para a PloS Neglected Tropical Diseases, escrevi sobre um cinturão de pobreza ignorado em meu país, os Estados Unidos da América, e sobre a incidência inaceitável de doenças entre nossos pobres de males como toxocaríase, cisticercose e toxoplasmose (1). De acordo com a Organização Pan-Americana de Saúde (OPAS – o escritório regional da Organização Mundial de Saúde nas Américas), a América Latina e a região do Caribe sofrem de bolsões de pobreza muito maiores e ainda com suas Doenças Tropicais Negligenciadas (DTNs) endêmicas (2). Enquanto procurava uma revisão sobre DTNs na América Latina, eu fiquei particularmente impressionado pela concentração desproporcional destas condições entre as pessoas pobres morando no Brasil. Apesar de não haverem gorilas nas salas do Brasil, acabo concluindo que as DTNs representam um grande tamanduá-bandeira (*Myrmecophaga tridactyla*, a maior espécie de tamanduá encontrada no Brasil e em outras localidades da américa tropical), que requer notificação, atenção e ação urgente.

De acordo com o Banco Mundial, 22% da população da América Latina e Caribe vivem com menos de 2 dólares por dia (3). Uma percentagem idêntica da população brasileira é também considerada miserável (4). Entretanto, com base no coeficiente de Gini, um índice que mede a desigualdade de renda ou a distribuição de riqueza, o Brasil, pontuando com 0.59, tem uma das maiores disparidades entre ricos e pobres do mundo (5). Uma das maneiras pelas quais tal desigualdade se manifesta é uma impressionante alta incidência de DTNs. Cerca de 40 milhões de brasileiros que vivem com menos de 2 dólares por dia (4) respondem por um terço de toda a população pobre vivendo na América Latina e região do Caribe (3), entretanto eles sofrem de forma desproporcional de DTNs. A tabela 1 mostra um sumário de DTNs no Brasil. Baseado em informações disponíveis em trabalhos publicados em revistas especializadas da área biomédica, bem como em websites de domínio público como OPAS e OMS (6-13), a maioria das DTNs da América Latina e Caribe atualmente ocorre no Brasil, incluindo virtualmente todos os casos de tracoma e hanseníase, além da maioria dos casos de ascaridíase, dengue, ancilostomíase, esquistossomose e leishmaniose visceral. Praticamente falando, tais dados mostram que a maioria dos 40 milhões de pobres do Brasil estão infectados com uma ou mais DTNs, especialmente ancilostomídeos (*Necator americanus*), ou nematelmintos (*Ascaris lumbricoides*), ou ambos. Para exemplificar, nosso grupo, em colaboração com o Centro de Pesquisas René Rachou da Fiocruz (Fundação Oswaldo Cruz), demonstrou recentemente que 68% da população de algumas comunidades rurais do estado de Minas Gerais estão infectadas com ancilostomídeos (14), causando anemia e malnutrição (15). Na mesma população carente, a presença de ancilostomídeos em seus intestinos promove a susceptibilidade a co-infecções com outros vermes, especialmente áscaris e esquistossoma (14).

Por todo o estado de Minas Gerais e nas circunvizinhanças do estado da Bahia, a ancilostomíase é conhecida como *amarelão*, referindo-se à cor amarelo-pálida da pele que resulta da infecção crônica. A ancilostomíase e outras DTNs são também importantes problemas de saúde pública entre o povo indígena do Brasil (16-19) e na população negra (20). Recentemente, um economista da Universidade de Chicago publicou que a infecção crônica com ancilostomídeo durante a infância repercute com uma redução de 43% nos ganhos salariais no futuro (21), demonstrando que a ancilostomíase representa uma importante causa pela qual pessoas pobres não conseguem escapar do círculo de pobreza. Este é o mesmo ancilostomídeo que exauria a energia do Jeca Tatu, o trabalhador popularizado pelo escritor brasileiro Monteiro Lobato no início do século XX. Uma importante característica de quase todas as DTNs é que elas não apenas ocorrem no cenário da pobreza, mas também promovem pobreza (22).

Em 2003, o presidente Luiz Inácio Lula da Silva lançou o *Fome Zero*, um ambicioso plano anti-pobreza de $500 milhões de dólares focalizado na malnutrição (23). Entretanto, como o Programa Alimentar Mundial já descobriu (24), na ausência de medidas conjuntas para o controle das DTNs, alimentar as crianças freqüentemente resulta em simplesmente alimentar os vermes primeiro. O controle conjunto de DTNs faria grande sentido uma vez que elas estão classificadas entre as medidas com melhor custo-benefício à saúde (22) e a uma taxa de 15-30% de retorno, além de uma medida anti-pobreza com ótimo custo-benefício (25, 26). Assim, o controle das DTNs representaria um dos mais eficientes e eficazes mecanismos para mudar a realidade de 40 milhões que estão na base da pobreza.

O Brasil tem todas as prerrogativas para lançar um esforço nacional articulado para solucionar uma das suas maiores disparidades na saúde. O país tem um presidente carismático que é comprometido com a população pobre, além de ter na FIOCRUZ e em outras agências do ministério da saúde e nas suas universidades alguns dos melhores especialistas em controle de doenças dos mundo (muitos deles participam do Conselho Editorial da *Plos Negleted Tropical Diseases*). Através dessas mesmas organizações, e de uma sofisticada infraestrutura biotecnológica de manufatura no Instituto Butantan (São Paulo) e Bio-manguinhos (Rio de Janeiro), o Brasil tem também a capacidade de inovar e produzir novas e melhores gerações de vacinas “anti-pobreza” para DTNs, incluindo vacinas para dengue, ancilostomídeo, leishmaniose, leptospirose, esquistossomose e febre amarela (27, 28). Junto com novas fundações comprometidas a resolver as disparidades na área da saúde e outras áreas nas Américas e algumas das pessoas mais ricas e influentes do mundo, o Brasil está numa excelente posição para estabelecer uma PPP (parceria público-privada) no controle, e em alguns casos, na eliminação das mais importantes DTNs. O Brasil já tomou a liderança na eliminação do seu problema de Doença de Chagas, que já foi uma das mais devastadoras DTNs no cone meridional da América do Sul (29), e tem feito grandes saltos no controle e eliminação da filariose linfática e da oncocercose (2, 12). Agora, assim como o Jeca Tatu, que uma vez curado da sua ancilostomíase galgou uma mudança de posição social, também uma nova PPP brasileira pode um dia eliminar suas DTNs de forma substancial para a redução da pobreza alcançando as Metas de Desenvolvimento do Milênio (Millennium Development Goals). À medida em que se aproxima a época do carnaval, é tempo de se lembrar dos 40 milhões de cidadãos mais pobres do Brasil. Nesse ínterim, PloS Negleted Tropical Diseases permanece comprometida em continuar recebendo e revendo trabalhos sobre DTNs no Brasil, e ajudando a facilitar a comunicação na comunidade científica brasileira.

Table 1. Burden of Neglected Tropical Diseases in Brazil

| Doença | Percentagem da incidência de doenças da América Latina que ocorre no Brasil | No. de casos estimado no Brasil | Referência |
| --- | --- | --- | --- |
| Tracoma | 97% | 1.06 milhões | 6 |
| Lepra | 93% | 44,436 novos casos (2006) | 7 |
| Esquistossomose | 83% | 1.5 milhões | 8 |
| Leishmaniose Visceral | 67% | 3,386 (2004) | 9 |
| Ancilostomíase | 65% | 32.3 milhões | 10 |
| Dengue | 63% | 346,471 casos descritos (2006) | 11 |
| Ascaridíase | 50% | 41.7 milhões | 10 |
| Leishmaniose cutânea | 46% | 28,375 (2004) | 9 |
| Tricuríase | 19% | 18.9 milhões | 10 |
| Filariose linfática | 8% | 60,000 | 12 |
| Oncocercose | 2% | 9,000 em risco | 2 |
| Leptospirose | Não determinado | Não determinado | 13 |

**Referências**

1. Hotez PJ. Neglected diseases of poverty in the “other America.” *PLoS Neglected Tropical Diseases* 2007; 3: in press.
2. Pan American Health Organization. *Health in the Americas 2007*, Volume I – Regional, Scientific and Technical Publication No. 622, Washington DC, pp. 176-82.
3. <http://siteresources.worldbank.org/DATASTATISTICS/Resources/lac_wdi.pdf>
4. <http://www.unaids.org/en/Regions_Countries/Countries/brazil.asp>
5. The World Bank. Inequality and Economic Development in Brazil, A World Bank Country Study. The International Bank for Reconstruction and Development/The World Bank 2004; Washington DC, Executive Summary, p. XVIII.
6. <http://www.who.int/globalatlas/dataQuery/reportData.asp?rptType=1>
7. <http://www.paho.org/English/AD/DPC/CD/lep-sit-reg-2007.pdf>
8. Steinmann P, Keiser J, Bos R, Tanner M, Utzinger J. Schistosomiasis and water resources development: systematic review, meta-analysis, and estimates of people at risk. *Lancet Infect. Dis* 2006; 6: 411-25.
9. Pan American Health Organization. *Health in the Americas 2007*, Volume II – Countries, Scientific and Technical Publication No. 622, Washington DC, pp. 131-53.
10. De Silva NR, Brooker S, Hotez PJ, Montresor A, Engels D, Savioli L. Soil-transmitted helminth infections: updating the global picture. *Trends Parasitol*  2003; 19: 547-51.
11. <http://www.paho.org/english/ad/dpc/cd/dengue-cases-2006.htm>
12. World Health Organization. Global Programme to Eliminate Lymphatic Filariasis. *Weekly Epidemiological Record* 2006; 81: 221-32.
13. McBride AJ, Athanazio DA, Reis MG, Ko AI. Leptospirosis. *Curr Opin Infect Dis*  2005; 18: 376-86.
14. Fleming FM, Brooker S, Geiger SM, Caldas IR, Correa-Oliveira R, Hotez PJ, Bethony JM. Synergistic associations between hookworm and other helminth species in a rural community in Brazil. *Trop Med Int Health*  2006; 11: 56-64.
15. Brooker S, Jardim-Botelho A, Quinnell RJ, Geiger SM, Caldas IR, Fleming F, Hotez PJ, Correa-Oliveira R, Rodrigues LC, Bethony JM. Age-related changes in hookworm infection, anaemia and iron deficiency in an area of high *Necator americanus* hookworm transmission in south-eastern Brazil. *Trans R Soc Trop Med Hyg* 2007; 101: 146-54.
16. Beltrame A, Scolari C, Torti C, Urbani C. Soil-transmitted helminth (STH) infections in an indigenous community in Ortigueria, Parana, Brazil and relationship with nutritional status. *Parassitologia* 2002; 44: 137-9.
17. Fontbonne A, Freese-De-Carvalho E, Acioli MD, Sa GA, Cesse EA. Risk factors for multiple intestinal parasites in an indigenous community of the state of Paernambuco, Braziil. *Cad Saude Publica* 2001, 17: 367-73.
18. Scolari C, Torti C, Beltrame A, Matteelli A, Castelli F, Gulletta M, Ribas M, Morana S, Urbani C. Prevalence and distribution of soil transmitted helminth infections in indigenous schoolchildren in Ortigueira, State of Parana, Brazil: implications for control. *Trop Med Int Health* 2000; 5: 302-7.
19. Ehrenberg JP, Ault SK. Neglected diseases of neglected populations: thinking to reshape the determinants of health in Latin America and the Caribbean. *BMC Public Health* 2005; 5: 119.
20. Lammie PJ, Lindo JF, Secor WE, Vasquez J, Ault SK, Eberhard ML. Elimination of lymphatic filariasis, onchocerciasis, and schistosomiasis from the Americas: breaking a historical legacy of slavery. *PLoS Negl Trop Dis* 2007; 1: e71.
21. Bleakley H. Disease and development: evidence from hookworm eradication in the American South. *Q J Econ* 2007; 122: 73-117.
22. Hotez PJ, Molyneux DH, Fenwick A, Kumaresan J, Ehrlich Sachs S, Sachs JD, Savioli L. Control of neglected tropical diseases. *N Engl J Med*  2007; 357: 1018-27.
23. BBC News World Edition. Brazil launches anti-poverty drive, 20 January 2003, <http://news.bbc.co.uk/2/hi/americas/2710797.stm>
24. <http://www.wfp.org/english/?ModuleID=137&Key=2333>
25. Molyneux DH. “Neglected” diseases but unrecognized successes – challenges and opportunities for infectious disease control. *Lancet*  2004; 364: 380-3.
26. Hotez PJ, Molyneux DH, Fenwick A, Ottesen E, Ehrlich Sachs S, Sachs JD. Incorporating a rapid-impact package for neglected tropical diseases with programs for HIV/AIDS, tuberculosis, and malaria. *PLoS Medicine*  2006; 3: e102.
27. Hotez PJ, Ferris M. The antipoverty vaccines. *Vaccine*  2006; 24: 5787-99.
28. Morel CM, Acharya T, Broun D, et al. Health innovation networks to help developing countries address neglected diseases. *Science*  2005; 309: 401-4.
29. Dias JC. Southern Cone Initiative for the elimination of domestic populations of Triatoma infestans and the interruption of transfusion of Chagas disease: historical aspects, present situation, and perspectives. *Mem Inst Oswaldo Cruz*  2007; [Epub ahead of print].
